# Effect of miR-412-5p–loaded exosomes in H9c2 cardiomyocytes via the MAPK pathway

**DOI:** 10.22038/IJBMS.2024.75590.16365

**Published:** 2024

**Authors:** Jin Hee Kim, June Hwan Lee

**Affiliations:** 1 Department of Anesthesiology and Pain Research Institute, Yonsei University College of Medicine, 50 Yonsei-ro, Seodaemun-gu, Seoul 03722, Republic of Korea; 2 Department of Energy Information Technology, Fareast University, 76-32, Daehak-gil, Gamgok-myeon, Eumseong-gun, Chungcheongbuk-do 27601, Republic of Korea

**Keywords:** Cardiomyocytes, Exosome, Inflammation, MAPK pathway, MiRNA-412-5p

## Abstract

**Objective(s):**

MicroRNAs (miRNAs) are small non-coding RNAs that function in all biological processes. Recent findings suggest that exosomes, which are small vesicles abundantly secreted by various cell types, can transport miRNAs to target cells. Here, we elucidated the effect of miRNA-loaded exosomes on lipopolysaccharide (LPS)-induced inflammation in H9c2 cardiomyocytes.

**Materials and Methods:**

Exosomes were isolated from mesenchymal stem cells (MSC) and loaded with miR-412-5p. Additionally, the effect of the miR-412-5p-loaded exosomes on LPS-induced inflammation in H9c2 cardiomyocytes was evaluated by assessing the levels of nitric oxide (NO), reactive oxygen species (ROS), and prostaglandin E2 (PGE2). The expression of cyclooxygenase-2 (COX-2), inducible nitric oxide synthase (iNOS), inflammatory cytokines, and mitogen-activated protein kinase (MAPK) signaling factors was evaluated using reverse transcription-quantitative PCR and western blotting.

**Results:**

miR-412-5p-loaded exosomes inhibited LPS-induced secretion of inflammatory mediators (NO, PGE2, and ROS), pro-inflammatory cytokines (*IL-1β* and *IL-6*), and COX-2 and iNOS expression. Additionally, miR-412-5p-loaded exosomes significantly decreased the expression of MAPK signaling molecules, including p-extracellular signal-regulated kinase (ERK), p-p38, and p-Jun kinase (JNK), in H9c2 cardiomyocytes.

**Conclusion:**

These findings showed that miR-412-5p-loaded exosomes ameliorated LPS-induced inflammation in H9c2 cardiomyocytes by inhibiting COX-2 and iNOS expression, inflammatory mediators, and pro-inflammatory cytokines via the MAPK pathway. The findings indicate that miR-412-5p-loaded exosomes may be effective for the prevention of myocardial injury.

## Introduction

MicroRNAs (miRNAs) are small non-coding RNAs of 19-25 nucleotides in length and function in all biological processes in organisms (1, 2). miRNAs are associated with various physiological processes, including human diseases, and are thus used as biomarkers for various diseases (2, 3). 

Exosomes are small vesicles secreted in abundance by various cell types (4) and involved in the delivery of specific contents to target cells, influencing biological processes without eliciting an immune response (5, 6). Previous studies have shown that mesenchymal stem cell (MSC)-derived exosomes exert beneficial effects in various diseases (7-9). Additionally, exosomes possess some advantages over synthetic nanoparticles, including the ability to pass through biological barriers and avoid the mononuclear phagocyte system, and have thus received considerable attention as potential carriers for genes and drugs (10-13). Moreover, some studies have shown that miRNA-loaded exosomes can induce apoptosis and angiogenesis in cancer cells (14-17). Overall, these findings indicate that exosomes can be used as miRNA delivery systems for the regulation of biological processes, including cardiac dysfunction.

Cardiac dysfunction is a common complication of severe sepsis and is associated with inflammatory responses (18-21). Specifically, myocardial dysfunction and heart failure can be caused by bacterial infections (22), and H9c2 cardiomyocytes have been extensively used to study cardiac dysfunction. Notably, H9c2 cells exhibit inflammatory response to LPS-induced stress (23), making them an ideal model for studying cardiac dysfunction (24-26). Although the therapeutic effects of miRNA-loaded exosomes have been reported in some diseases, studies on the effects of miRNA-loaded exosomes on myocardial dysfunction are limited. Here, we elucidated the effects and potential mechanisms of miR-412-5p-loaded exosomes in cardiac dysfunction using an LPS-stimulated H9c2 cardiomyocyte model. It is anticipated that this study will promote the clinical adoption of exosomes as miRNA carriers for the treatment of several diseases, including myocardial dysfunction.

## Materials and Methods

***Cell culture***

H9c2 cardiomyoblasts were purchased from the Korea Cell Line Bank (Seoul, Korea) and maintained in Dulbecco’s modified Eagle’s medium (DMEM) supplemented with 10% fetal bovine serum (FBS, HyClone, Logan, UT, USA) and 1% penicillin-streptomycin. The murine embryonic mesenchymal stem cell line C3H/10T1/2 was purchased from the Korea Cell Line Bank (Seoul, Korea) and cultured in RPMI-1640 medium containing 10% FBS and 1% penicillin-streptomycin. Prior to the experiments, the cells were seeded in 96-well plates at a density of 1.5×10^4^ cells per well for the cell proliferation assay, or in 6-well plates at a density of 5×10^6^ cells per well for all other assays. Each experiment was performed in triplicate. 

***Isolation of MSC-exosomes***

For isolation of MSC-exosomes, C3H/10T1/2 cells (passage 9) were cultured in a serum-free medium for 24 hr. The culture medium was harvested and filtered through a 0.2-μm syringe filter (Sartorius, Sottingen, Germany) to remove microvesicles. C3H/10T1/2 cell culture supernatants were centrifuged at 300× g and 2,000× g for 10 min at 4 ^°^C to remove cell debris. Exosomes were isolated from the MSC culture medium using a commercial exosome isolation kit (Total Exosome Isolation kit, Invitrogen™), according to the manufacturer’s protocol. In brief, 5 ml of the exosome precipitation solution provided in the kit was added to 10 ml of the supernatant, and the mixture was incubated overnight at 4 ^°^C. The samples were then centrifuged at 10,000× g for 1 hr at 4 ^°^C; the resulting pellet, which contained the exosomes, was obtained. For the subsequent experiments, H9c2 cells were treated with MSC-exosomes at a concentration of 3×10^9 ^particles. The isolated MSC-exosomes were stored at -70 ^°^C until use. 

***Characterization of extracted exosomes***

Exosomes were purified, and scanning electronic microscopy (SEM) and nanoparticle tracking analysis (NTA) using the NanoSight NS300 (Malvern, UK) were employed to confirm the quality of the exosome preparations. Western blot analyses were performed with specific antibodies (CD9 and CD63) against exosome biomarker proteins. 

***Generation of miR-142-5p-loaded exosomes***

We transfected miR-412-5p (100 nM) into the MSC-derived exosomes using an ExoFect siRNA/miRNA Transfection Kit (System Biosciences, Mountain View, CA, USA), according to the manufacturer’s instructions. miR-412-5p was incubated with Exo-Fect transfection reagent at room temperature for 15 min. Then, we added exosomes (100 μg in a total volume of 100 μl) and incubated the mixture again at 37 ^°^C for 1 hr. The samples were then transferred to a spin column and incubated with gentle rotation at room temperature for 10 min. The samples were centrifuged at 1,000× g for 30 sec, collected, and then stored at -70 ^°^C until use.

***LPS treatment***

H9c2 cardiomyoblasts were treated with different LPS concentrations (0, 500, 1000, and 1500 ng/ml) for 24 hr. We found that LPS significantly inhibited cell viability after treatment at 1000 and 1500 ng/ml. Combining our results with those of a previous study (27), we selected an LPS concentration of 1000 ng/ml for the subsequent experiments.

***Cell viability assay***

H9c2 cardiomyoblasts were seeded into 96-well plates (1.5×10^4^ cells/well) for 24 hr before the experiment. Cells were treated with the exosomes or miR-412-5p-loaded exosomes for 24 hr after LPS stimulation. Cell viability was determined using the 3-(4,5-dimethylthiazol-2-yl)-5-(3-carboxymethoxyphenyl)-2-(4-sulfophenyl)-2H-tetrazolium (MTS) proliferation assay (CellTiter 96®AQueous Cell Proliferation Assay kit; Promega Corporation, Madison, WI, USA), following the manufacturer’s instructions. In brief, 20 μl MTS was added into each well and incubated for 1-4 hr at 37 ^°^C. The optical density of each well was measured at 490 nm. 

***Measurement of nitrite (NO) production***

H9c2 cardiomyoblasts were seeded into 12-well plates (6×10^5^ cells/well) for 24 hr before the experiment. Cells were treated with exosomes or miR-412-5p-loaded exosomes after LPS stimulation. The culture media were collected and centrifuged at 10,000 rpm at 4 ^°^C for 10 min. The amount of nitrite was determined using the Griess Reagent System (Promega). Briefly, NO levels in the culture supernatants were measured by adding 50 μl of sulfanilamide solution and 50 μl of 0.1% N-naphthyl-ethylenediamine (NED) solution to 50 μl of culture supernatant in each well and incubating for 15 min at room temperature in the dark. The absorbance at 540 nm was then measured using a microplate reader. A standard curve was used to calculate the concentration of NO. The curve was derived from the NaNO_2_ standard solution that was treated in the same manner as the culture supernatants.

***Measurement of reactive oxygen species (ROS) production***

 H9c2 cardiomyoblasts were seeded into a 96-well plate (1.5×10^4^ cells/well) for 24 hr before the experiment. Cells were treated with the exosome or miR-412-5p-loaded exosomes after LPS stimulation. To measure ROS levels in cells, 10 μM of DCFDA-Cellular ROS assay buffer (Abcam, Cambridge, UK) was added to the cell culture media, and the plate was incubated at 37 ^°^C for 30 min. Cell fluorescence was measured using a microplate reader after excitation at 485 nm and emission at 535 nm.

***Quantitative polymerase chain reaction (qPCR)***

Total RNA was extracted from cells using the RNeasy Mini Kit (Qiagen, Valencia, CA, USA) and reverse-transcribed using CycleScript RT PreMix (Bioneer, Daejeon, South Korea). We performed qRT-PCR on a system using SYBR Green PCR Master Mix (Applied Biosystems Inc., Foster City, CA, USA) and specific primers. The mRNA levels of* COX-2 *and* iNOS *were normalized to the level of *GAPDH*. 

Total RNA from MSC-exosomes was purified using the miRNeasy mini kit (Qiagen, Hilden, Germany) according to the manufacturer’s instructions. The RNA concentration was quantified using a NanoDrop (ND-2000) spectrophotometer (Thermo Fisher Scientific). Complementary DNA was synthesized from 500 ng of total RNA using the miScript II RT Kit. Quantification of miRNAs was performed using the miScript SYBR Green PCR Kit (Qiagen) on the ABI StepOne Real-Time PCR system according to the manufacturer’s instructions (Applied Biosystems, Foster City, CA, USA). The primer sequences are listed in Table 1.

***Western blot analysis***

Briefly, H9c2 cells were harvested and homogenized in a lysis buffer (Cell Signaling Technology, Inc., Danvers, MA, USA). Protein concentration was determined using the Pierce BCA Protein Assay Kit (cat. no. 23225; Thermo Fisher Scientific, Inc., Waltham, MA, USA). Proteins were separated on 12 and 8% sodium dodecyl sulfate–polyacrylamide gels, transferred to nitrocellulose membranes (Thermo Fisher Scientific, Inc.), then blocked with 5% skim milk for 1 hr at room temperature, and incubated with primary antibodies overnight at 4 ^°^C. After three washes with Tris-buffered saline containing 0.1% Tween 20, the membranes were incubated with secondary horseradish peroxidase-conjugated anti-IgG antibody. Western blot bands were observed using Pierce Pierce-enhanced chemiluminescence substrate (Thermo Fisher Scientific, Inc.). Band intensities were determined using the ImageJ software (version 1.29x, National Institutes of Health, Bethesda, MD, USA), and the band intensities were normalized to the intensity of the housekeeping gene (GAPDH). 

***Enzyme-linked immunosorbent assay (ELISA)***

Prostaglandin E2 (PGE2) levels were quantified using a PGE_2_ ELISA kit (R&D Systems, Minneapolis, MN, USA), according to the manufacturer’s instructions. 

***Statistical analysis***

Quantitative data are presented as mean±standard error (SE). All statistical analyses were performed using the SPSS software (version 22.0; IBM Corp., Armonk, NY, USA), and multiple comparisons were performed. Statistical significance was set at *P*<0.05, and all experiments were performed at least three times. 

## Results

***Characterization and identification of miR-412-5p-loaded exosomes***

**Figure 1:**
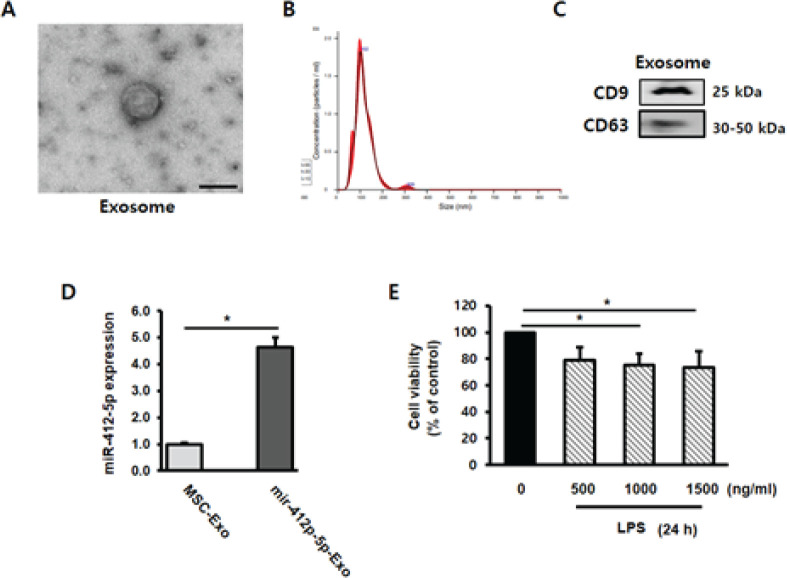
Characterization and validation of mesenchymal stem cell (MSC)-derived exosomes and miR-412-5p-loaded exosomes

 C3H/10T1/2 cell culture media were collected and purified to obtain MSC-derived exosomes. Electronic microscopy (EM) showed that the diameter of the exosomes was approximately 100 nm (Figure 1A). Additionally, nanoparticle tracking analysis (NTA) was used to determine the size distribution of the exosomes (Figure 1B). Moreover, western blotting analysis detected the expression of the exosomal markers CD9 and CD63, confirming that the particles were exosomes (Figure 1C).

To determine the loading efficiency of miR-412-5p into exosomes, miR-412-5p expression levels were measured using qPCR. Compared with that in unloaded MSC-derived exosomes, miR-412-5p expression was up-regulated in miR-412-5p-loaded exosomes (Figure 1D). 

 To determine the effect of inflammation on LPS treatment, H9c2 cells were treated with 0, 500, 1000, and 1500 ng/ml of LPS for 24 hr. Compared with the control group, cell viability was significantly reduced (*P*<0.05) after treatment with 1000 and 1500 ng/ml of LPS (Figure 1E). Therefore, cells were treated with 1000 ng/ml LPS for 24 hr in subsequent experiments. 

***miR-412-5p-loaded exosomes decreased secretion of inflammatory cytokines in LPS-induced H9c2 cardiomyocytes***

**Figure 2:**
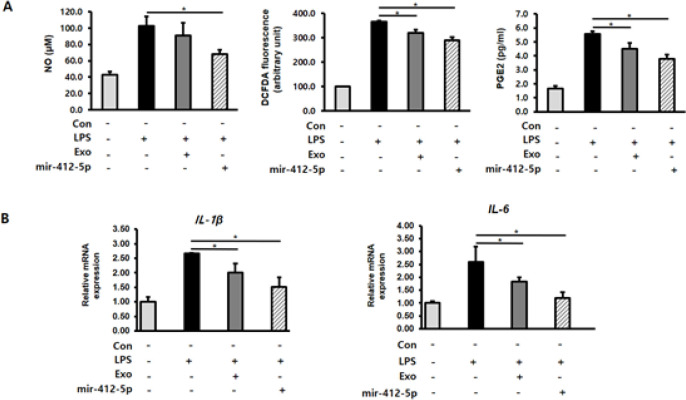
MiR-412-5p loaded exosomes decrease oxidative stress and IL-1β and IL-6 expression in lipopolysaccharide (LPS)-treated H9c2 cardiomyocytes

 Inflammatory mediators such as NO and PGE2 have been reported to play important roles in the inflammatory process (28, 29). To determine the effect of miR-412-5p-loaded exosomes on inflammation, we examined the NO, ROS, and PGE2 levels of H9c2 cells treated with the loaded exosomes after LPS stimulation. Compared with the LPS-stimulated group, treatment with miR-412-5p-loaded exosomes significantly suppressed (*P*<0.05) LPS-induced increase in NO, ROS, and PGE2 levels (Figure 2). Additionally, the effect of miR-412-5p-loaded exosomes on inflammatory cytokines, including *IL-1β *and* IL-6*, was determined using qPCR. Compared with the LPS-stimulated group, treatment with miR-412-5p-loaded exosomes significantly suppressed (*P*<0.05) the LPS-induced increase in *IL-1β *and* IL-6* mRNA expression (Figure 2). 

***miRNA 412-5p-loaded exosomes decreased COX-2 and iNOS expression in LPS*** ***‐*** ***stimulated H9c2 cardiomyocytes***

**Figure 3:**
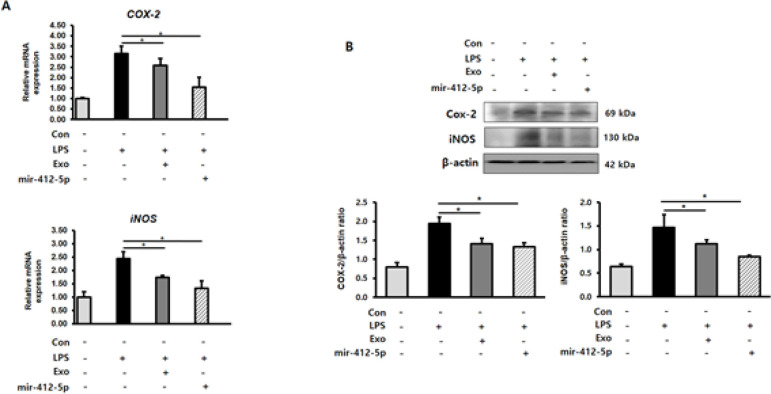
MiR-412-5p loaded exosomes decrease COX-2/iNOS expression in lipopolysaccharide (LPS)-treated H9c2 cardiomyocytes

 We examined the effect of miR-412-5p-loaded exosomes on LPS-induced expression of COX-2 and iNOS in H9c2 cardiomyocytes using qPCR and western blot analysis. Compared with the LPS-stimulated group, treatment with miR-412-5p-loaded exosomes significantly suppressed (*P*<0.05) the LPS-induced increase in COX-2 and iNOS expression in H9c2 cardiomyocytes at both mRNA and protein levels (Figure 3A, B). 

***miRNA 412-5p-loaded exosomes regulated LPS-stimulated H9c2 cardiomyocytes via the MAPK pathway***

**Figure 4:**
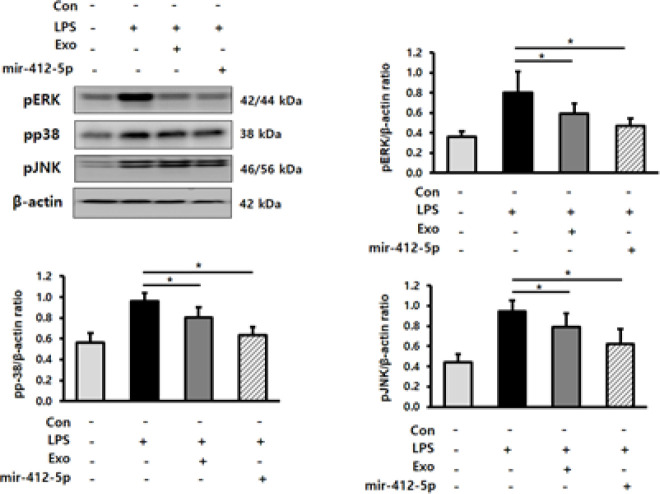
MiR-412-5p loaded exosomes regulate the MAPK pathway in lipopolysaccharide (LPS)-treated H9c2 cardiomyocytes

 To elucidate the mechanism of action of miR-412-5p, we examined the expression of MAPK signaling molecules, including p-ERK, p-p38, and p-JNK, using western blot analysis. Compared with the LPS-treated group, p-ERK, p-p38, and p-JNK protein levels were significantly down-regulated (*P*<0.05) in LPS-stimulated H9c2 cardiomyocytes treated with miR-412-5p-loaded exosomes (Figure 4).

## Discussion

In this study, we investigated the protective effects and mechanism of miR-412-5p-loaded exosomes against inflammatory injury in H9c2 cardiomyocytes. Notably, miR-412-5p-loaded exosomes exerted anti-inflammatory effects by suppressing LPS-induced inflammation, including ROS production and iNOS and COX-2 expression. Additionally, the results showed that miR-412-5p-loaded exosomes may be effective against inflammatory injury via regulation of MAPK pathways, suggesting its potential as a nanotherapeutics.

Several studies have shown that LPS can induce ROS generation, followed by activation of various transcription factors and intracellular signaling cascades, leading to the synthesis of inflammatory mediators (30-32). In the cardiovascular system, LPS has been shown to induce pro-inflammatory cytokine production and dysfunction (30, 31).

Various functions can be regulated by miR-412-5p, including vasculature formation (33). Additionally, Yang *et al*. reported a decrease in miR-412-5p expression in a model mimicking early cerebral ischemia (34). In the present study, miR-412-5p-loaded exosomes inhibited inflammatory mediators, including NO and PGE2, suppressed ROS production, and markedly reduced the levels of pro-inflammatory cytokines, including IL‐1β and IL-6, in LPS-stimulated H9c2 cardiomyocytes. Overall, these results suggested that miR-412-5p-loaded exosomes inhibited ROS and inflammatory cytokine production. 

LPS has been reported to increase the expression of COX-2 and prostaglandin during myocardial inflammation (35-37). Consistent with these findings, COX‐2 mRNA expression and protein levels were up-regulated in LPS-stimulated H9c2 cardiomyocytes in the present study. However, treatment with miR-412-5p-loaded exosomes effectively suppressed the LPS-induced increase in COX‐2 level, which may decrease prostaglandin secretion. A previous study showed that LPS increased NO levels in cells by enhancing iNOS expression (38). Similarly, the present study showed that LPS treatment increased iNOS protein levels in H9c2 cardiomyocytes; however, treatment with miRNA412-5p-loaded exosomes inhibited the LPS-induced increase in iNOS levels. 

We verified whether miR-412-5p exerted its anti-inflammatory effect in H9c2 cardiomyocytes via the MAPK pathway. The MAPK pathway is an important signaling pathway in inflammatory disorders (39, 40), and regulates the expression of inflammatory cytokines against various pathogens (41). Moreover, the MAPK pathway regulates inflammatory mediators, including COX-2, iNOS, and IL-1β. Previous studies have reported that ERK and p38 MAPK are involved in regulating the activities of IL-1 and COX-2 (42-44). Additionally, the MAPK pathway has been shown to participate in the iNOS expression pathway (45). miR-412-5p-loaded exosomes decreased LPS-induced activation of p-ERK, p-p38, and p-JNK in H9c2 cardiomyocytes, suggesting that the MAPK signaling pathway may be involved in the anti-inflammatory effect of miR-412-5p-loaded exosomes in H9c2 cardiomyocytes.

**Table 1:** Sequence of primers used in real-time quantitative PCR for gene expression

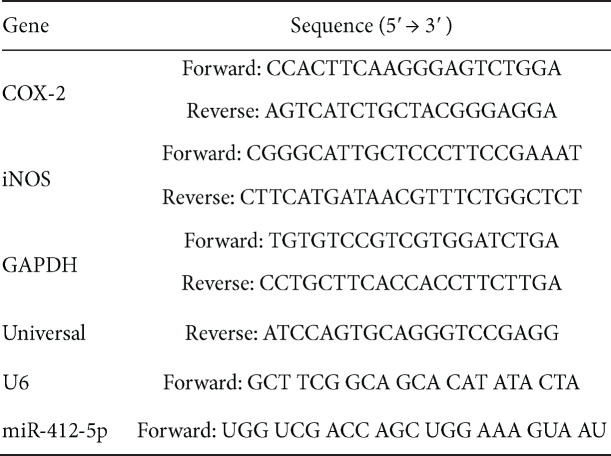

(A) Representative scanning electron microscopy (SEM) images of the exosomes isolated from MSC. Scale bar=100 nm. (B) Nanoparticle tracking analysis (NTA) of exosome size in MSC culture media (n=3). (C) Protein levels of the exosomal markers CD9 and CD63 in exosomes (n=3). (D) To determine the effect of miR-412-5p transfection on MSC-exosomes, the levels of miR-412-5p were measured using qPCR. (E) H9c2 cardiomyocytes were treated with 0, 500, 1000, and 1500 ng/ml lipopolysaccharides (LPS) for 24 hr. Data are presented as the mean±standard deviation

(A) H9c2 cardiomyocytes treated with miR-412-5p-loaded exosomes showed decreased NO, PGE2, and ROS levels compared with the LPS-treated group. (B) IL-1β and IL-6 mRNA expression in cardiomyocytes treated with miR-412-5p-loaded exosomes was determined using a quantitative polymerase chain reaction. Data are presented as the mean±standard deviation. *P<0.05

(A) IL-1β and IL-6 mRNA expression in H9c2 cardiomyocytes treated with miR-412-5p-loaded exosomes following LPS stimulation was analyzed using quantitative polymerase chain reaction. (B) IL-1β and IL-6 protein levels in H9c2 cardiomyocytes treated with miR-412-5p-loaded exosomes were analyzed using western blot analysis. Data are presented as the mean±standard deviation. *P<0.05

Western blotting analysis was performed to determine the expression of phosphorylated ERK, p38, and JNK protein levels in H9c2 cardiomyocytes treated with miR-412-5p-loaded exosomes, following LPS stimulation. Data are presented as the mean±standard deviation. *P<0.05

## Conclusion

The findings of this study indicate that miR-412-5p-loaded exosomes ameliorated LPS-induced inflammation in H9c2 cardiomyocytes by inhibiting COX-2 and iNOS expression, NO and ROS levels, and inflammatory cytokines via the MAPK pathway. Overall, these findings suggest that miR-412-5p-loaded exosomes may be effective in preventing myocardial injury.

## Authors’ Contributions

JH K and JH L designed the experiments; JH K and JH L conducted the experiments and wrote the paper; JH K and JH L approved the final version to be published.

## Conflicts of Interest

The authors declare no conflicts of interest regarding the publication of this paper. 
